# Adherence to Low Carbohydrate Diet in Relation to Chronic Obstructive Pulmonary Disease

**DOI:** 10.3389/fnut.2021.690880

**Published:** 2021-08-03

**Authors:** Hanieh Malmir, Shokouh Onvani, Mohammad Emami Ardestani, Awat Feizi, Leila Azadbakht, Ahmad Esmaillzadeh

**Affiliations:** ^1^Obesity and Eating Habits Research Center, Endocrinology and Metabolism Molecular Cellular Sciences Institute, Tehran University of Medical Sciences, Tehran, Iran; ^2^Food Security Research Center, Isfahan University of Medical Sciences, Isfahan, Iran; ^3^Department of Community Nutrition, School of Nutrition and Food Science, Isfahan University of Medical Sciences, Isfahan, Iran; ^4^Division of Pulmonology, Department of Internal Medicine, School of Medicine, Isfahan University of Medical Sciences, Isfahan, Iran; ^5^Department of Epidemiology and Biostatistics, Endocrinology and Metabolism Research Center, Isfahan University of Medical Sciences, Isfahan, Iran; ^6^Department of Community Nutrition, School of Nutritional Science and Dietetics, Tehran University of Medical Sciences, Tehran, Iran; ^7^Diabetes Research Center, Endocrinology and Metabolism Clinical Sciences Institute, Tehran University of Medical Sciences, Tehran, Iran

**Keywords:** chronic obstructive pulmonary disease, low-carbohydrate high-fat diet, case-control study, pulmonary function, FFQ

## Abstract

**Purpose:** Data on the link between adherence to low-carbohydrate diet (LCD) and odds of chronic obstructive pulmonary disease (COPD) are scarce. The current study aimed to investigate the relation between adherence to LCD and COPD in Iranian adults.

**Methods:** In this hospital-based case-control study, we enrolled 84 newly-diagnosed COPD patients and 252 age and sex matched healthy controls in Alzahra University Hospital, Isfahan, Iran. COPD was defined based on findings of spirometry test (forced expiratory volume in 1 s (FEV1)/forced vital capacity (FVC) < 70% or FEV1 < 80%). Dietary intakes of study participants were assessed using the validated Block-format 168-item FFQ. Data on potential confounders were also collected through the use of a pre-tested questionnaire.

**Results:** Mean age of cases and controls were 57.7 and 55.07 years, respectively. Adherence to LCD was inversely associated with odds of COPD (0.35; 95% CI: 0.16-0.75). This inverse association did not alter after controlling for age, sex, and energy intake (0.42; 95% CI: 0.19-0.93). Adjustments for other potential confounders, including dietary intakes, smoking, and educational status, did not affect these findings; such that those in the highest quintile of LCD score were 64% less likely to have COPD than those in the lowest quintile (OR: 0.36; 95% CI: 0.13-0.99).

**Conclusion:** We found an inverse association between adherence to LCD and odds of COPD. The association remained statistically significant even after taking other potential confounders, including socioeconomic characteristic and dietary intakes into account.

## Introduction

Chronic obstructive pulmonary disease (COPD) is an inflammatory lung disease characterized by progressive airflow limitation (1). It is the fourth leading cause of death in the world. More than 210 million people globally and 15 million people in US are affected (2). The World Health Organization (WHO) has predicted that COPD will become the third most common cause of death in the world by 2030 (3). The symptoms often appear when significant lung damages have occurred. The main symptom is a daily cough and mucus production (4).

Active and passive exposure to tobacco smoke is the most significant risk factor for COPD. In addition to air pollution, occupational pollutants, age, and genetics, several nutritional factors have also been investigated in relation to COPD (5). For instance, fruit and vegetable consumption, fish intake, vitamin D and C supplementation, and low intake of cured meats have been linked with a lower risk of COPD (6–9). Due to the different respiratory quotients (RQs) of carbohydrates, proteins and fats, inappropriate long term intake of these macronutrients might affect lung health. Consumption of high-fat low-carbohydrate diets, less tha <130 grams of carbohydrate per day, was associated with easier breathing in COPD patients (10–12). However, findings from clinical trials cannot be easily generalized to daily routine life due to the use of a high-dose intervention in a short time. To prevent COPD in the community, observational studies which focus on normal routine life are required. Consumption of a low carbohydrate diet in epidemiologic studies was inversely associated with obesity, cardiovascular disease and diabetes (13–17); however, the link between adherence to a low carbohydrate diet in usual life and risk of COPD was not investigated.

Assessment of the contribution of low carbohydrate diet to chronic conditions is particularly relevant for Middle East countries, where people usually consume high amounts of carbohydrates and low amounts of dietary proteins (18, 19). Although the quantity of dietary fat intake in these countries is not so high, the quality of dietary fats needs further attention. On the other hand, dietary intake of antioxidants, a well-known contributor to lung health, in these countries is low. Therefore, this study aimed to investigate the relation between adherence to a low carbohydrate diet and odds of COPD in Iranian adults.

## Methods

### Participants

This hospital-based case-control study was carried out on 84 newly-diagnosed COPD patients and 252 healthy controls [non-COPD patients] in Alzahra University Hospital, Isfahan, Iran, in 2015. Cases were individuals with forced expiratory volume in 1 s (FEV1)/forced vital capacity (FVC) < 70% or FEV1 < 80%. Controls were individuals without a history of COPD who were hospitalized in the same hospital. Case and control groups were individually matched in terms of age (±5), and sex. Individuals older than 30 years of age and those whom COPD diagnosis was based on physician diagnosis and spirometry test were not included in the case group. Participants were excluded if they had stroke, dementia, or any condition that would preclude the possibility for an interview. Moreover, other chronic diseases including chronic liver cirrhosis, renal failure, uncontrolled thyroid disease, inflammatory bowel disease, rheumatoid arthritis, severe heart failure, cachexia, cancer in the past 3 years, chronic infections (HIV, tuberculosis, etc.), systematic period of treatment by steroids drugs for a long time, and other pulmonary problems such as fibrosis were considered as non-inclusion criteria due to their effects on patients' dietary patterns or inability of these people to respond to questions. All cases and controls provided their written informed consent. The study was ethically approved by the Medical Ethics Committee of the Tehran University of Medical Sciences, Tehran, Iran.

### Assessment of Dietary Intakes

Usual dietary intakes of participants over the past year were assessed using a validated 168-item FFQ. The FFQ consisted of 168 food items with a standard serving size for each food item (20). A trained interviewer administered the FFQ through face to face interviews. All reported consumptions were converted to grams per day using household measures (21). Subsequently, daily intakes of energy and nutrients were computed for each person using the US Department of Agriculture food composition database using Nutritionist IV software (www.worldcat.org) that was modified for Iranian foods (22).

To assess the adherence to the low carbohydrate diet (LCD), we used a validated method which described below and divided the study participants into deciles of fat, protein and carbohydrate intakes, expressed as a percentage of energy (23, 24). For fat and protein, highest intake received 10 points and lowest intake received 1 points. For carbohydrate, the order of the received points was reverse; those with the lowest carbohydrate intake received 10 points and those with the highest carbohydrate intake received 1 points. Then, the points for each of the three macronutrients were summed to create the overall diet score, which ranged from 3 (the lowest fat and protein intake and the highest carbohydrate intake) to 30 (the highest protein and fat intake and the lowest carbohydrate intake). Therefore, the higher the score, the more closely the participant's diet followed the pattern of a low-carbohydrate diet. Cut points were taken from control groups.

### Assessment of Pulmonary Function

A trained technician assessed pulmonary function with spirometry test and calculated FEV1, FVC, and FEV1/FVC. Other respiratory symptoms, including chronic cough, sputum production, and breathlessness, were assessed. Chronic cough refers to coughing for more than 3 weeks (25). Sputum production for more than 3 months in 2 consecutive years is an epidemiologic definition of sputum production (26, 27). Breathlessness was assessed using a visual analog scale. A visual analog scale is a 100-mm horizontal line with descriptive words on both sides for individuals to explain their breathlessness rate using picture observations (28).

### Assessment of Other Variables

Required information about age, gender, marital status, education level, cigarette smoking (current smoker, ex-smoker, never smoker), familial history of pulmonary disorders, and history of drug and supplement use were collected through the use of a pre-tested questionnaire. Body weight was quantified by digital scale to the nearest 100 g with minimal clothes and bare feet. Height was measured without shoes with shoulders in a normal position. BMI was calculated as weight in kilograms divided by height in square meters. The long form of the International Physical Activity Questionnaire (IPAQ) was administered to examine participants' daily physical activity (29). Physical activity was calculated based on metabolic equivalent for task (MET), number of days per week, and amount of time per day (minutes) and was finally expressed as MET.min/week.

### Statistical Methods

Participants were categorized into quintiles based on LCD score. General characteristics and dietary intakes of participants across quintiles of LCD score were compared using one-way ANOVA for continues variables and chi-square tests for categorical variables. The association between LCD score and COPD was assessed by using logistic regression in different models. Model 1 adjusted for age, sex, and energy intake (kcal/d). Additional adjustment was conducted for university education (yes/no), and smoking status (yes/no). Finally, we adjusted the analysis for dietary intakes of red and processed meats, whole grain, and sugar-sweetened beverages. All confounders were chosen based on previous publications (30–34). The statistical analyses were carried out by using SPSS version 21. *P*-values were considered significant at <0.05.

## Results

General characteristics of cases and controls are shown in Table 1. Cases were more likely to be married, smoker, employed, physically active and have family history of pulmonary disease and exposure to industrial air pollution. As expected, FEV1, FVC, and FEV1/FVC were significantly higher in cases than controls.

**Table 1:** Characteristics of patients with COPD and control subjects.

	**Cases (** ***n*** **=** **84)**		**Control (** ***n*** **=** **252)**		
	**%**	**Mean + SD**	**%**	**Mean + SD**	***P*-value**
Age (Year)		57.07 ± 12.47		55.05 ± 12.34	0.197
Sex (Male)	89.3		89.3		0.999
Married (%)	90.5		87.6		0.030
University Education (%)	6		9.1		0.362
Employment status (%)	21.4		12.9		<0.0001
Weight (Kg)		71.0 ± 14.5		73.9 ± 12.7	0.077
Height (m)		166.6 ± 9.2		169.0 ± 9.2	0.040
BMI (Kg/m^2^)		25.6 ± 4.8		25.9 ± 3.8	0.550
Physical activity (MET/week)		5285 ± 8097		11759 ± 6307	<0.0001
History of pulmonary disease (%)	39.3		19.8		0.006
Air pollution in habitat (%)					<0.0001
Urban	41.7		78		
Rural	41.7		13.9		
Industrial	16.7		8.1		
Hookah use (%)	100		39.5		<0.0001
Cigarette smoking (%)					<0.0001
Current smoker	57.1		44.8		
Former smoker	0.0		22.0		
Never smoker	31.0		7.8		
Smoke exposure (%)	16.7		12.3		0.431
FEV1		55.2 ± 18.6		95.0 ± 12.5	<0.0001
FVC		71.2 ± 17.9		92.6 ± 13.3	<0.0001
FEV1/FVC		62.7 ± 9.3		82.5 ± 6.1	<0.0001

*COPD, chronic obstructive pulmonary disease; BMI, body mass index; FEV1, forced expiratory volume in 1 s; FVC, forced volume capacity*.

*P-values were calculated by ANOVA for continuous variables and chi-square test for categorical variables*.

Dietary intakes of cases and controls were presented in Table 2. Patients with COPD had lower intakes of whole grains, red and processed meats, and sugar-sweetened beverages than controls. In addition, they had greater intakes of energy, carbohydrate, cholesterols, sodium, magnesium, Vitamin K, and dietary fibers than controls.

**Table 2:** Mean dietary intakes among patients with COPD and control subjects.

	**Cases** **(*n* = 84)**	**Control** **(*n* = 252)**	
	**Mean + SD**	**Mean + SD**	***P*-value**
**Food groups (g/d)**			
Whole grains	49.3 ± 91.9	125.8 ± 115.9	<0.0001
Fruit	362.8 ± 214.1	368.7 ± 180.4	0.811
Vegetables	352.9 ± 151.0	340.1 ± 173.6	0.554
Low-fat dairy products	397.6 ± 237.6	352.1 ± 170.6	0.065
Legume and nuts	43.9 ± 38.2	46.9 ± 28.6	0.458
Red meat/processed meat	69.8 ± 37.9	53.6 ± 41.3	0.002
Sugar-sweetened beverages	45.9 ± 76.7	63.5 ± 71.3	0.061
**Nutrients**			
Energy (Kcal/d)	2,882 ± 781	2,624 ± 682	0.004
Carbohydrate (g/d)	462 ± 137	415 ± 128	0.005
Protein (g/d)	95 ± 25	110 ± 159	0.390
Fat (g/d)	78 ± 31	70 ± 35	0.087
Cholesterol (mg/d)	290 ± 151	245 ± 117	0.005
Sodium (mg/d)	3,565 ± 1,108	4,308 ± 1,570	<0.0001
Magnesium (mg/d)	309 ± 106	429 ± 154	<0.0001
Potassium (mg/d)	3,901 ± 1,324	4,299 ± 2,709	0.197
Calcium (mg/d)	1,246 ± 413	1,187 ± 459	0.305
Vitamin E (g/d)	7.5 ± 4	7.2 ± 5	0.677
Folate (ug/d)	384 ± 139	386 ± 110	0.310
Vitamin C (mg/d)	144 ± 70	143 ± 61	0.920
Vitamin K (mg/d)	124 ± 106	390 ± 286	<0.0001
Dietary fiber (g/d)	19 ± 7	22 ± 10	0.021

Comparing across quintiles of LCD score, we found that participants with the highest LCD score were more likely to be physically active, employed, smoker, expose to air pollution, have lower mean weight and BMI and less likely to use hookah than those with the lowest score (Table 3). Looking at dietary intakes, we observed that those with the greatest adherence to LCD had higher intakes of low fat dairy, whole grains, red and processed meats, proteins, fats, cholesterol, magnesium, vitamin E and vitamin K and lower intakes of energy and carbohydrates than those with the lowest adherence (Table 4).

**Table 3:** Characteristics of study participants among quintiles of LCD score.

	**Q1**	**Q2**	**Q3**	**Q4**	**Q5**	***P***
Age (Year)	56.1 ± 11.9	56.4 ± 12.2	55.1 ± 13.6	54.3 ± 11.8	55.4 ± 13.1	0.897
Sex (Male) (%)	93.0	89.7	86.7	90.4	90.0	0.801
Married (%)	93.0	89.7	88.3	90.4	84.7	0.657
University Education (%)	4.7	7.4	3.3	9.6	16.7	0.051
Employment status (%)	15.9	12.5	21.8	13.7	10.2	<0.0001
Weight (Kg)	75.5 ± 16.1	73.9 ± 10.8	68.8 ± 12.4	74.6 ± 12.9	72.2 ± 10.5	0.033
Height (m)	169.2 ± 8.8	167.3 ± 7.9	167.9 ± 10.5	170.0 ± 9.4	167.6 ± 10.2	0.446
BMI (Kg/m^2^)	26.4 ± 5.3	26.5 ± 3.9	24.3 ± 3.1	25.9 ± 3.4	25.8 ± 3.4	0.020
Physical activity (MET/Min/week)	7,974 ± 8,339	9,525 ± 8,488	11,068 ± 6,019	11,402 ± 5,387	11,343 ± 7,130	0.029
History of pulmonary disease (%)	28.6	32.5	25.0	46.2	23.8	0.658
Air pollution in habitat (%)						0.002
Urban	52.4	62.9	72.7	88.2	75.5	
Rural	31.7	25.8	21.8	9.8	12.2	
Industrial	15.9	11.3	5.5	2.0	12.2	
Hookah use (%)	85.4	67.2	38.2	27.5	44.9	<0.0001
Cigarette smoking (%)						<0.0001
Current smoker	44.6	39.7	50	46.2	61.8	
Former smoker	34.9	44.4	39.3	44.2	27.3	
Never smoker	20.5	15.9	10.7	9.6	10.9	
Smoke exposure (%)	14.3	17.5	10.0	7.7	19.0	0.837
FEV1	72.7 ± 26.4	74.5 ± 23.7	79.8 ± 31.1	76.8 ± 22.1	74.1 ± 24.5	0.868
FVC	80.5 ± 19.5	83.4 ± 15.9	82.7 ± 23.8	84.6 ± 20.0	78.7 ± 19.7	0.844
FEV1/FVC	71.9 ± 13.2	72.5 ± 11.2	73.9 ± 15.4	72.0 ± 10.3	71.7 ± 13.5	0.981

*COPD, chronic obstructive pulmonary disease; BMI, body mass index; FEV1, forced expiratory volume in 1 s; FVC, forced volume capacity*.

*P-values were calculated ANOVA test for continuous variables and chi-square test for categorical variables*.

**Table 4:** Mean dietary intakes of study participants among quintiles of LCD score.

	**Q1**	**Q2**	**Q3**	**Q4**	**Q5**	***P***
**Food groups (g/d)**						
Whole grains	59 ± 96	110 ± 115	150 ± 134	139.5 ± 116	83 ± 87	<0.0001
Fruit	393 ± 237	391 ± 149	324 ± 139	334 ± 187	383 ± 190	0.129
Vegetables	377 ± 193	346 ± 124	346 ± 125	318 ± 150	318 ± 153	0.235
Low-fat dairy products	335 ± 195	348 ± 164	367 ± 171	370 ± 165	451 ± 238	0.013
Legume and nuts	44 ± 27	46 ± 29	43 ± 36	45.5 ± 25	49.5 ± 28	0.786
Red meat/processed meat	58 ± 32	64.5 ± 36	45 ± 25	48 ± 27	66 ± 47	0.002
Sugar-sweetened beverages	56 ± 91	56 ± 64	46 ± 47	74 ± 70	62 ± 81	0.380
**Nutrients**						
Energy (kcal/d)	2,959 ± 832	2,829 ± 621	2,467 ± 598	2,576 ± 651	2,461 ± 655	<0.0001
Carbohydrate (g/d)	500 ± 167	453 ± 95	407 ± 98	406 ± 95	347 ± 96	<0.0001
Protein (g/d)	89 ± 24	96 ± 20	94 ± 22	101 ± 23	103 ± 26	0.004
Fat (g/d)	69 ± 28	77 ± 32	58 ± 19	68.5 ± 27	81 ± 31	<0.0001
Cholesterol (mg/d)	244 ± 105	276 ± 168	219 ± 75	257 ± 110	304 ± 145	0.004
Sodium (mg/d)	3,951 ± 1,452	4,327 ± 1,441	4,118 ± 1,397	4,208 ± 1,459	4,208 ± 1,643	0.604
Magnesium (mg/d)	327 ± 110	390 ± 151	429 ± 145	474 ± 163	431 ± 152	<0.0001
Potassium (mg/d)	3,915 ± 1,292	3,928 ± 847	3,985 ± 1,013	4,160 ± 1,281	4,290 ± 1,134	0.257
Calcium (mg/d)	1,129 ± 348	1,165 ± 295	1,209 ± 475	1,216 ± 465	1,241 ± 378	0.450
Vitamin E (mg/d)	7.3 ± 3.6	8.2 ± 4.5	5.7 ± 2.9	6.1 ± 3.0	8.8 ± 8.1	0.001
Folate (um/d)	380 ± 135	387 ± 115	352 ± 92	368 ± 111	376 ± 108	0.511
Vitamin C (mg/d)	146 ± 69	153 ± 50	133 ± 57	140 ± 68	148 ± 65	0.405
Vitamin K (mg/d)	177 ± 184	270 ± 269	384 ± 274	458 ± 282	422 ± 296	<0.0001
Dietary fiber (g/d)	20 ± 7.5	22 ± 8	20 ± 7	21.5 ± 10	23.5 ± 13	0.179

Multivariable-adjusted odds ratios and 95% confidence intervals for COPD across quartiles of LCD score are shown in Table 5. Adherence to LCD was inversely associated with odds of COPD (0.35; 0.16-0.75, *P* = 0.007). This inverse association did not alter after controlling for age, sex, and energy intake (0.42; 0.19-0.93, *P* = 0.031). Adjustments for other potential confounders, including dietary intakes, smoking and educational status, did not affect these findings; such that those in the highest quintile of LCD score were 64% less likely to have COPD than those in the lowest quintile (OR: 0.36; 95% CI: 0.13-0.99, *P* = 0.045).

**Table 5:** Multivariable-adjusted odds ratios for COPD across quintiles of LCD scores.

	**Q1**	**Q2**	**Q3**	**Q4**	**Q5**	***P*-trend**
Crude	1	0.62 (0.32-1.21)	0.21 (0.09-0.50)	0.18 (0.07-0.47)	0.35 (0.16-0.75)	<0.0001
Model 1	1	0.64 (0.33-1.27)	0.25 (0.10-0.62)	0.21 (0.08-0.55)	0.42 (0.19-0.93)	0.002
Model 2	1	0.82 (0.38-1.77)	0.26 (0.09-0.68)	0.28 (0.08-0.66)	0.41 (0.17-0.99)	0.005
Model 3	1	1.03 (0.42-2.51)	0.31 (0.10-0.93)	0.27 (0.09-0.85)	0.36 (0.13-0.99)	0.005

*Adjusted odds ratio is results from logistic regression*.

*Model 1: adjusted for age, sex, and energy intake*.

*Model 2: model 1 + education level and smoking status*.

*Model 3: model 2 + whole grain, Sugar-sweetened beverages and processed meat*.

## Discussion

We found an inverse association between adherence to LCD and odds of COPD. The association remind statistically significant even after taking other potential confounders, including socioeconomic characteristic and dietary intakes into account. This study is the first to examine the association between adherence to LCD and odds of COPD in case-control study.

COPD has a high mortality rate in the world. This condition appears when significant lung damages have occurred (5). Dietary intakes are among other significant factors contributing to COPD. In a randomized double blind study, consumption of a low-carbohydrate high-fat diet resulted in a significantly lower production of CO2 (35). Patients with COPD and hypercapnia fed low, moderate, and high carbohydrate diets to determine the effects on metabolic and ventilatory values. Individuals in the low-carbohydrate diet had lower production of CO2 (*P* < 0.002), respiratory quotient (*P* < 0.001), and arterial Pco2 (*P* < 0.05) compared with those in other groups. Another randomized study demonstrated that consumption of a high-fat low-carbohydrate diet affected pulmonary function. In that study individuals in the experimental group had decreased lung function measurements and increased forced expiratory volume compared with those in the control group (36). Our findings in the context of an observational study also revealed an inverse relationship between adherence to LCD and odds of COPD in Iranian adults. Although findings from clinical trials are much stronger than those from case-control studies, reaching a similar finding in observational studies indicate that these findings could be generalized to the normal life of people.

Low-carbohydrate high-fat diet can affect pulmonary function through its influence on respiratory quotient (37, 38). As we all know, RQ for carbohydrate (1.0) is higher than that for protein (0.8) and fat (0.7). At the same volume of oxygen input, CO2 production would be higher with a high-carbohydrate diet, while consumption of a high-fat low-carbohydrate diet can result in decreased RQ and pulmonary function (39). On the other hand, consumption of a high-fat very low-carbohydrate diet, called as ketogenic diets, might favorably affect the lung inflammation through production of beta-hydroxybutyrate. Consumption of ketogenic diet would result in expending high amounts of fats as a fuel, rather than glucose, and this would in turn produce ketone bodies at the amount of higher than CO2. High production of ketone bodies has been associated with suppressed nucleotide-binding oligomerization domain-like receptor 3 (NLRP3), which is the main inflammatory driver in COPD (40, 41). One of the points that should be considered in interpreting the findings of this article is the relationship between shortness of breath and carbohydrate consumption. COPD patient and others who suffer from shortness of breath and chronic cough might be easier to consume lower amount of carbohydrate (42, 43). In fact, the relation between carbohydrate intake and COPD is bilateral and this confounder must be considered.

This study has several strengths. It is the first observational study that assessed the relationship between consumption of LCD and risk of COPD in the Middle East. We controlled for a wide range of confounders in the present study to reach an independent association. Cases and controls were matched in terms of age and sex to avoid their probable contributing effect on COPD. Moreover, spirometry testing was done for all participants to ensure correct classification of study participants in terms of COPD status. This study has some limitations as well. Because of the case-control design, selection and recall biases cannot be avoided. Previous epidemiological studies have shown that cases generally recall their usual past dietary intakes better than controls. We enrolled controls from individuals who were hospitalized in the same hospitals. In hospital-based case-control studies, different patterns of referring cases and controls might result in a greater selection bias. In the current study, FFQ was used for assessing dietary intakes. As we all know, the accuracy of reported dietary intakes by FFQ is lower than those obtained from dietary records or recalls. Therefore, the possibility of misclassification of participants in terms of dietary intakes cannot be excluded. Furthermore, observational studies cannot reveal a causal relationship. Therefore, prospective studies are required to confirm these findings.

## Conclusion

In conclusion, our findings suggested an inverse association between consumption of LCD diet and odds of COPD, even after taking potential confounders into account, in a group of adult individuals. However, further studies are required to confirm these findings.

**Ethical Approval and Consent to participate: 393881**

## Data Availability Statement

The original contributions presented in the study are included in the article/supplementary material, further inquiries can be directed to the corresponding author/s.

## Ethics Statement

The studies involving human participants were reviewed and approved by Medical Ethics Committee of the Tehran University of Medical Sciences, Tehran, Iran. The patients/participants provided their written informed consent to participate in this study.

## Author Contributions

HM, SO, MA, AF, and AE designed the research. HM and AE conducted the research, performed statistical analysis, and wrote the paper. AE had responsibility for final content. All authors read and approved the final manuscript.

## Conflict of Interest

The authors declare that the research was conducted in the absence of any commercial or financial relationships that could be construed as a potential conflict of interest.

## Publisher's Note

All claims expressed in this article are solely those of the authors and do not necessarily represent those of their affiliated organizations, or those of the publisher, the editors and the reviewers. Any product that may be evaluated in this article, or claim that may be made by its manufacturer, is not guaranteed or endorsed by the publisher.

## References

[1] Bracke KR Brusselle GG. Chapter 97—Chronic obstructive pulmonary disease. In: Mestecky J Strober W Russell MW Kelsall BL Cheroutre H Lambrecht BN, editors. Mucosal Immunology (Fourth Edition). Boston, MA: Academic Press (2015). p. 1857-66.

[2] MacNee W. Chapter 41—Chronic obstructive pulmonary disease: epidemiology, pathophysiology, and clinical evaluation. In: Spiro SG Silvestri GA Agustí A, editors. Clinical Respiratory Medicine (Fourth Edition). Philadelphia, PA: W.B. Saunders (2012). p. 531-52.

[3] WHO. COPD Predicted to be Third Leading Cause of Death in 2030. (2008). Available online at: https://www.who.int/gard/news_events/World_Health_Statistics_2008/en/ (accessed December, 2020).

[4] Clinic M. COPD 2017. Available online at: https://www.mayoclinic.org/diseases-conditions/copd/symptoms-causes/syc-20353679 (accessed January, 2021).

[5] WHO. Chronic Obstructive Pulmonary Disease (COPD) 2017. Available online at: https://www.who.int/news-room/fact-sheets/detail/chronic-obstructive-pulmonary-disease-(copd) (accessed January, 2021).

[6] Jiang R Paik DC Hankinson JL Barr RG. Cured meat consumption, lung function, and chronic obstructive pulmonary disease among United States adults. Am J Respir Crit Care Med. (2007) 175:798-804. 10.1164/rccm.200607-969OC 17255565 PMC1899290

[7] Kaluza J Larsson SC Orsini N Linden A Wolk A. Fruit and vegetable consumption and risk of COPD: a prospective cohort study of men. Thorax. (2017) 72:500. 10.1136/thoraxjnl-2015-207851 28228486

[8] Tsiligianni IG van der Molen T. A systematic review of the role of vitamin insufficiencies and supplementation in COPD. Respir Res. (2010) 11:171. 10.1186/1465-9921-11-171 21134250 PMC3016352

[9] Varraso R Barr RG Willett WC Speizer FE Camargo CA Jr. Fish intake and risk of chronic obstructive pulmonary disease in 2 large US cohorts. Am J Clin Nutr. (2015) 101:354-61. 10.3945/ajcn.114.094516 25646333 PMC4307205

[10] McClave SA Lowen CC Kleber MJ Wesley McConnell J Jung LY Goldsmith LJ. Clinical use of the respiratory quotient obtained from indirect calorimetry. JPEN J Parenter Enteral Nutr. (2003) 27:21-6. 10.1177/014860710302700121 12549594

[11] Florian IS. Nutrition and COPD - Dietary Considerations for Better Breathing. (2009). Available online at: https://www.todaysdietitian.com/newarchives/td_020909p54.shtml (accessed December, 2020).

[12] Scoditti E Massaro M Garbarino S Toraldo DM. Role of diet in chronic obstructive pulmonary disease prevention and treatment. Nutrients. (2019) 11:1357. 10.3390/nu11061357 31208151 PMC6627281

[13] Arora SK McFarlane SI. The case for low carbohydrate diets in diabetes management. Nutr Metab. (2005) 2:16. 10.1186/1743-7075-2-16 16018812 PMC1188071

[14] Share Investigators S-A. Protein intake is inversely associated with abdominal obesity in a multi-ethnic population. J Nutr. (2005) 135:1196-201. 10.1093/jn/135.5.1196 15867303

[15] Merchant AT Vatanparast H Barlas S Dehghan M Shah SMA De Koning L. Carbohydrate intake and overweight and obesity among healthy adults. J Am Diet Assoc. (2009) 109:1165–72. 10.1016/j.jada.2009.04.002 19559132 PMC3093919

[16] Hu T Bazzano LA. The low-carbohydrate diet and cardiovascular risk factors: evidence from epidemiologic studies. Nutr Metab Cardiovasc Dis. (2014) 24:337–43. 10.1016/j.numecd.2013.12.008 24613757 PMC4351995

[17] Ebbeling CB Feldman HA Klein GL Wong JMW Bielak L Steltz SK. Effects of a low carbohydrate diet on energy expenditure during weight loss maintenance: randomized trial. BMJ. (2018) 363:k4583. 10.1136/bmj.k4583 30429127 PMC6233655

[18] Musaiger A. Food Consumption Patterns in the Eastern Mediterranean Region. Arab Center for Nutrition, Manama (2011). p. 1–101.33143163

[19] Aghayan M Asghari G Yuzbashian E Mahdavi M Mirmiran P Azizi F. Secular trend in dietary patterns of Iranian adults from 2006 to 2017: Tehran lipid and glucose study. Nutr J. (2020) 19:110–8. 10.1186/s12937-020-00624-x 33010805 PMC7533031

[20] Mirmiran P Esfahani FH Mehrabi Y Hedayati M Azizi F. Reliability and relative validity of an FFQ for nutrients in the Tehran lipid and glucose study. Public Health Nutr. (2010) 13:654–62. 10.1017/S1368980009991698 19807937

[21] Ghaffarpour MH-RA Kianfar H. The Manual for Household Measures, Cooking Yields Factors and Edible Portion of Foods [in Farsi]. Tehran: Keshaverzi Press (1999).

[22] Haytowitz DB Lemar LE Pehrsson PR Exler J Patterson KK Thomas RG. USDA National Nutrient Database for Standard Reference, Release 24. (2011). Available online at: http://www.ars.usda.gov/Services/docs.htm?docid=8964 (accessed December, 2020).

[23] Mirmiran P Asghari G Farhadnejad H Eslamian G Hosseini-Esfahani F Azizi F. A low carbohydrate diet is associated with a reduced risk of metabolic syndrome in Tehranian adults. Int J Food Sci Nutr. (2016) 68:1–8. 10.1080/09637486.2016.1242119 27718762

[24] Halton T Willett W Liu S Manson J Albert C Rexrode K Hu F. Low-carbohydrate-diet score and the risk of coronary heart disease in women. N Engl J Med. (2006) 355:1991–2002. 10.1056/NEJMoa055317 17093250

[25] Stulbarg MAL. Manifestation of respiratory disease. In: Broaddus VC Ernst J King TE Sarmiento KF, editors. Murray and Nadel's Textbook of Respiratory Medicine. Philadelphia, PA: Elsevier Saunders (2015). p. 497-514.

[26] Pauwels RA Buist AS Calverley PM Jenkins CR Hurd SS. Global strategy for the diagnosis, management, and prevention of chronic obstructive pulmonary disease. NHLBI/WHO Global Initiative for Chronic Obstructive Lung Disease (GOLD) Workshop summary. Am J Respir Crit Care Med. (2001) 163:1256–76. 10.1164/ajrccm.163.5.2101039 11316667

[27] Celli BR MacNee W. Standards for the diagnosis and treatment of patients with COPD: a summary of the ATS/ERS position paper. Eur Respir J. (2004) 23:932–46. 10.1183/09031936.04.00014304 15219010

[28] Mahler DA Weinberg DH Wells CK Feinstein AR. The measurement of dyspnea. Contents, interobserver agreement, and physiologic correlates of two new clinical indexes. Chest. (1984) 85:751–8. 10.1378/chest.85.6.751 6723384

[29] Moghaddam MHB Aghdam F Asghari Jafarabadi M Allahverdipour Hamid Nikookheslat S. The Iranian version of international physical activity questionnaire (ipaq) in Iran: content and construct validity, factor structure, internal consistency and stability. World Appl Sci J. (2012) 18:1073–80. 10.5829/idosi.wasj.2012.18.08.754

[30] Machado MC Krishnan JA Buist SA Bilderback AL Fazolo GP Santarosa MG. Sex differences in survival of oxygen-dependent patients with chronic obstructive pulmonary disease. Am J Respir Crit Care Med. (2006) 174:524–9. 10.1164/rccm.200507-1057OC 16778158

[31] Suissa S Sotgiu G Brusasco V. Observational studies in COPD: summary of guidance for authors. COPD: J Chronic Obstructive Pulmonary Dis. (2018) 15: 415-7. 10.1080/15412555.2018.1555234 30822246

[32] Salari-Moghaddam A Milajerdi A Larijani B Esmaillzadeh A. Processed red meat intake and risk of COPD: a systematic review and dose-response meta-analysis of prospective cohort studies. Clin Nutr. (2019) 38:1109–16. 10.1016/j.clnu.2018.05.020 29909249

[33] Tabak C Smit HA Heederik D Ocké MC Kromhout D. Diet and chronic obstructive pulmonary disease: independent beneficial effects of fruits, whole grains, and alcohol (the MORGEN study). Clin Exp Allergy. (2001) 31:747–55. 10.1046/j.1365-2222.2001.01064.x 11422134

[34] DeChristopher LR Uribarri J Tucker KL. Intake of high fructose corn syrup sweetened soft drinks is associated with prevalent chronic bronchitis in U.S. Adults, ages 20-55 y. Nutr J. (2015) 14:107. 10.1186/s12937-015-0097-x 26474970 PMC4609055

[35] Angelillo VA Bedi S Durfee D Dahl J Patterson AJ O'Donohue WJ Jr. Effects of low and high carbohydrate feedings in ambulatory patients with chronic obstructive pulmonary disease and chronic hypercapnia. Ann Intern Med. (1985) 103:883–5. 10.7326/0003-4819-103-6-883 3933397

[36] Tümer G Mercanligil SM Uzun O Aygün C. The effects of a high-fat, low-carbohydrate diet on the prognosis of patients with an acute attack of chronic obstructive pulmonary disease. Turkiye Klinikleri J Med Sci. (2009) 29:895–904.

[37] Marques-Vidal P Pecoud A Hayoz D Paccaud F Mooser V Waeber G. Prevalence and characteristics of vitamin or dietary supplement users in Lausanne, Switzerland: the CoLaus *study*. Eur J Clin Nutr. (2009) 63:273–81. 10.1038/sj.ejcn.1602932 17940542

[38] Cai B Zhu Y Ma Y Xu Z Zao Y Wang J. Effect of supplementing a high-fat, low-carbohydrate enteral formula in COPD patients. Nutrition (Burbank, Los Angeles County, Calif). 2003 Mar;19(3):229-32. PubMed PMID: 12620524. Epub 2003/03/07. eng. 10.1016/S0899-9007(02)01064-X 12620524

[39] Patel H Kerndt CC Bhardwaj A. Physiology, Respiratory Quotient. Treasure Island, FL: StatPearls (2020). 30285389

[40] Yang W Ni H Wang H Gu H. NLRP3 inflammasome is essential for the development of chronic obstructive pulmonary disease. Int J Clin Exp Pathol. (2015) 8:13209–16. 26722520 PMC4680465

[41] Youm YH Nguyen KY Grant RW Goldberg EL Bodogai M Kim D. The ketone metabolite beta-hydroxybutyrate blocks NLRP3 inflammasome-mediated inflammatory disease. Nat Med. (2015) 21:263–9. 10.1038/nm.3804 25686106 PMC4352123

[42] Mann D Bass P. How to Improve Beathing With COPD. Everyday Health. (2013). Available online at: https://www.everydayhealth.com/copd-awareness-guide/improve-breathing-with-copd.aspx

[43] Cleveland Clinic Medical Professional. Nutritional Guidelines for People with COPD. (2018). Available online at: https://my.clevelandclinic.org/health/articles/9451-nutritional-guidelines-for-people-with-copd (accessed January, 2021).

